# Surface Waters and Urban Brown Rats as Potential Sources of Human-Infective *Cryptosporidium* and *Giardia* in Vienna, Austria

**DOI:** 10.3390/microorganisms9081596

**Published:** 2021-07-27

**Authors:** Silvia Cervero-Aragó, Amélie Desvars-Larrive, Gerhard Lindner, Regina Sommer, Iveta Häfeli, Julia Walochnik

**Affiliations:** 1 Institute for Hygiene and Applied Immunology, Medical University of Vienna, 1090 Vienna, Austria; gerhard.lindner@meduniwien.ac.at (G.L.); regina.sommer@meduniwien.ac.at (R.S.); 2 Interuniversity Cooperation Centre Water & Health, Vienna, Austria; 3 Veterinary Public Health and Epidemiology Unit, University of Veterinary Medicine, 1210 Vienna, Austria; Amelie.Desvars@vetmeduni.ac.at; 4 VetFarm, University of Veterinary Medicine, 1210 Vienna, Austria; 5 Complexity Science Hub Vienna, 1080 Vienna, Austria; 6 Institute of Specific Prophylaxis and Tropical Medicine, Medical University of Vienna, 1090 Vienna, Austria; iveta.haefeli@meduniwien.ac.at (I.H.); julia.walochnik@meduniwien.ac.at (J.W.)

**Keywords:** *Giardia*, *Cryptosporidium*, *Eimeria*, brown rats, *Rattus norvegicus*, One Health, zoonosis

## Abstract

*Cryptosporidium* and *Giardia* are waterborne protozoa that cause intestinal infections in a wide range of warm-blooded animals. Human infections vary from asymptomatic to life-threatening in immunocompromised people, and can cause growth retardation in children. The aim of our study was to assess the prevalence and diversity of *Cryptosporidium* and *Giardia* in urban surface water and in brown rats trapped in the center of Vienna, Austria, using molecular methods, and to subsequently identify their source and potential transmission pathways. Out of 15 water samples taken from a side arm of the River Danube, *Cryptosporidium* and *Giardia* (oo)cysts were detected in 60% and 73% of them, with concentrations ranging between 0.3–4 oocysts/L and 0.6–96 cysts/L, respectively. *Cryptosporidium* and *Giardia* were identified in 13 and 16 out of 50 rats, respectively. *Eimeria*, a parasite of high veterinary importance, was also identified in seven rats. Parasite co-ocurrence was detected in nine rats. Rat-associated genotypes did not match those found in water, but matched *Giardia* previously isolated from patients with diarrhea in Austria, bringing up a potential role of rats as sources or reservoirs of zoonotic pathogenic *Giardia*. Following a One Health approach, molecular typing across potential animal and environmental reservoirs and human cases gives an insight into environmental transmission pathways and therefore helps design efficient surveillance strategies and relevant outbreak responses.

## 1. Introduction

Even though the Danube River is the second largest river in Europe, information regarding the prevalence of protozoan enteric pathogens in its water is scarce. In a comprehensive study, Kirschner et al. [1] reported that human-associated faecal pollution is a crucial problem throughout the Danube River basin, posing a threat to all types of water uses. *Cryptosporidium* and *Giardia* are parasitic protozoa responsible for diarrheal diseases in humans and other animals worldwide [2]. Although infections caused by the two parasites are underreported, the prevalence of *Cryptosporidium* among the world’s population is estimated to range between 3–5% while prevalence is approximately 10% for *Giardia* [3]. In the USA these parasites are responsible for 30,000 cases of diarrhea every year [3].

The two parasites have similar life cycles, characterized by an environment-resistant infective stage, the *Cryptosporidium* oocysts and *Giardia* cysts, which initiate infection following ingestion, even at a very low dose [4]. A large number of *Cryptosporidium* and *Giardia* infections remain asymptomatic [5]. Symptomatic infections progress with abdominal pain and watery, usually self-limiting, diarrhea that can be life-threatening in children and immunocompromised people [6]. Moreover, *Cryptosporidium* infection can lead to long-term sequelae and has been associated with human colon cancer [7] while *Giardia* infections cause nutrient malabsorption and growth retardation in children regardless of the severity of the symptoms [8]. In industrialized countries, giardiasis is referred to as a re-emerging infectious disease because of its increasingly recognized role in outbreaks of diarrheal disease in daycare centers and in water- and foodborne outbreaks [9].

The transmission of both pathogens occurs via the fecal-oral route, through the ingestion of contaminated food or water. Both parasites are considered waterborne pathogens and are responsible for a great number of outbreaks associated with consumption of contaminated water or ingestion during recreational water activities (reviewed in [10]). Among the 381 waterborne parasitic protozoan outbreaks that occurred between 2011–2016 worldwide, *Cryptosporidium* was responsible of 63% of them, whereas 37% of them were associated with *Giardia* [3]. Identification at the species and genotype/assemblage level of environmental *Cryptosporidium* and *Giardia* is crucial to assessing the risk for human infections and potential infection sources [11].

Up until now, through molecular methods, 42 species of the genus *Cryptosporidium* are recognized and more than 50 genotypes remain to be formally described, due to the lack of biological and/or genetic data [12]. Among them *C. hominis* and *C. parvum* are responsible for the majority of infections in humans [11]. However, almost 20 other species and genotypes of *Cryptosporidium* have been reported in humans (reviewed by [12]), among them *Cryptosporidium* species that also infect rodents.

Although the taxonomy of the genus *Giardia* it is still under discussion, there is a global agreement that *G. duodenalis* is the species responsible for the majority of human infections [4]. *G. duodenalis*, has been divided into eight assemblages (A–H), each with varying host specificities [4,13,14]. Assemblages A and B are responsible for 37% and 58% of human infections, respectively [15]; however, both have very broad host ranges including dogs, cats, livestock and wildlife. While assemblage B is usually associated with prolonged and severe cases of diarrhea and is considered more pathogenic, assemblage A seems to be generally more abundant in the environment [8]. Assemblages C and D are typically found in canids like dogs, assemblage E in cattle and other livestock, assemblage F in cats, assemblage G in rats and assemblage H in seals [4,8,13]. Sporadic cases of human infection with assemblages C, D, E and F have been reported, providing evidence of zoonotic transmission [15].

In 2018, an estimated 55.3% of the world’s population lived in urban settlements. By 2030, urban areas are projected to house 60% of people globally [16]. Thus, urban public health is becoming a discipline of critical importance. This also includes the transmission of waterborne pathogens via urban surface waters. Norway or brown rats (*Rattus norvegicus*) and black rats (*Rattus rattus*) are among the most ubiquitous urban species [17], and are considered successful urban exploiters due to their high ability to adapt and thrive in close contact with humans [18,19]. This close proximity between rats and humans provides opportunity for the cross-species transmission of pathogens. Thus, rats are considered carriers and sometimes reservoirs of zoonotic pathogens, e.g., *Leptospira* spp., *Rickettsia typhi, Bartonella* spp., *Streptobacillus moniliformis* and hantavirus, posing a public health concern [20,21,22]. Studies of *Cryptosporidium* in rats conducted in both urban and rural environments in Australia, China, Japan, the United Kingdom and New Zealand have reported prevalence ranging from 2% to 49% [23]. Recent molecular characterization of *Cryptosporidium* that infect mice and rats identified the following species/genotypes: zoonotic *C. parvum*, *C. meleagridis*, *C. muris*, potentially zoonotic *C. tyzzeri*, and host adapted species such as mouse genotype II and rat genotypes I, II, III and IV [23,24]. Moreover, wild rodents can be infected with different *Giardia* species, including *G. muris* and various assemblages of *G. duodenalis* (assemblages A, B and the rodent-exclusive G) [25] with a prevalence ranging from 22.2–100% [26].

The dynamics of infections at the animal-human interface are determined by changes in host populations such as rat and pathogen prevalence and diversity, abundance, spatial distribution and contact rates within the rats–humans–pathogens system. Moreover, site-specific abiotic factors [19] as well as factors related to anthropogenic activities can also impact infection dynamics. Therefore, local studies are needed in order to accurately assess the risk and adapt relevant preventive strategies.

Using a One Health approach, this study aimed to (i) assess for the first time the prevalence of parasitic protozoa such as *Cryptosporidium* and *Giardia* in urban surface water such as the Danube Canal, a side-arm of the River Danube that flows through the city center of Vienna, Austria, and in urban brown rats trapped in the city center of Vienna, (ii) determine the diversity and zoonotic potential of the aforementioned parasites by identifying species, genotypes, and assemblages, and (iii) assess the potential role of the River Danube and urban brown rats as sources and/or reservoirs of *Cryptosporidium* and *Giardia* in the study area.

## 2. Materials and Methods

### 2.1. Trapping

*R. norvegicus* were trapped between March and June 2017 at two sites in the city of Vienna, Austria, highly frequented by humans: at a promenade along the Danube Canal (mean coordinates of the trapped rats: 16.36540, 48.22633 decimal degrees (D.D.)) and at Karlsplatz (16.37044, 48.20363 D.D.), a tourist attraction in the city. Detailed information on the trapping method used can be found elsewhere [27].

Rats were identified at the species level based on morphological characteristics and named with codes AD31 to AD84. No feces samples were obtained for rats AD53, AD60, AD65 and AD75. For each animal, morphological data were recorded such as sex, body mass (g), body length (nose to anus, mm) and sexual maturity. Sexual maturity was assessed for males when rats developed seminal vesicles and had scrotal (vs. inguinal) testes. According to Vadell et al. [28], females were assessed as sexually mature when showing a distinct uterus blood supply, placental scars or presence of embryos. Feces were collected from the rectum and stored in 96% ethanol until DNA extraction.

### 2.2. Cryptosporidium and Giardia Quantification in Water Samples

Surface water samples from the Danube Canal (48.211826, 16.383592), a side arm of the River Danube that flows through the city center of Vienna, were analyzed monthly for the presence of *Giardia* and *Cryptosporidium* between May 2019 and September 2020, except during March and April due to SARS-CoV-2 derived lockdown (*n* = 15). Water volumes of 5–15 L were enumerated following the flat membrane method described in ISO 15553 [29]. Briefly, after filtration, 142 mm cellulose acetate membranes with pore size 1.2 µm were placed in stomacher bags and transported to the lab. Particles on the membranes were scraped with a cell scraper and recovered using 50 mL of Glycine 1M (Sigma Aldrich, Steinheim, Germany) buffer at pH 5.5, followed by an incubation of 10 min in the Stomacher Lab-Blender 400 and 5 min in an ultrasound bath. The contents of the bags were then placed into 50 mL tubes and centrifuged at 1550× *g* for 15 min. Supernatants were discarded and pellets were resuspended in 2 mL of ultrapure water. One mL of the suspension was used for identification of the parasites by molecular methods (explained below). The remaining 1 mL was then used for immunomagnetic separation of *Giardia* and *Cryptosporidium* using the Dynabeads GC Combo kit (Life Technologies, Oslo, Norway). Concentrates were stained with the EasyStain kit (Biopoint Pty. Ltd., Belrose, Australia) and quantified as described in [30]. After implementation, tests were performed to determine the recovery efficiency of the used enumeration method when spiking surface water samples. For that purpose, reference materials *G. muris* H3 and *C. parvum* (Waterborne Inc, New Orleans, LA, USA) were used. As water matrixes we used several samples from surface waters with different turbidity ranging from 1.7–70 NTUs (Supplementary Material Table S2). Recovery efficiencies were 47 ± 27% and 58 ± 28% for *Cryptosporidium* oocysts and *Giardia* cysts, respectively. The theoretical limit of detection (LOD) of the flat membrane method varied according to the volume of water analyzed. For a 10 L water sample, the LOD was 0.4 (oo)cyst/L.

### 2.3. DNA Extraction

Approximately 200 mg of rat stool was dried using silica for 48 h. DNA extraction was performed using the QIAamp^®^ Fast DNA Stool Mini Kit (Qiagen, Hilden, Germany), according to the manufacturer’s instructions. DNA extraction from water samples was carried out with the DNeasy^®^ Power Soil Kit (Qiagen, Hilden, Germany), according to the manufacturer’s instructions, using 200 µL of concentrated water samples.

### 2.4. Identification of Cryptosporidiumand Giardia Species and Genotypes

For identification below the genus level, all samples were subjected to two independent nested PCRs, specific to *Cryptosporidium* and *Giardia* respectively.

For identification of *Cryptosporidium*, the nested PCR described by [31] was used, targeting a fragment of the 18S rDNA gene. The PCR consisted of 45 cycles at 94 °C for 30 s, 58 °C for 30 s and 72 °C for 30 s. The conditions for the secondary nested PCR were identical to those of the primary PCR. *Giardia* was identified by nested PCR targeting a fragment of the triosephosphate isomerase (*tpi*) gene [9]. Conditions for the primary and secondary nested PCRs were identical and consisted of 35 cycles at 94 °C for 45 s, 50 °C for 45 s and 72 °C for 1 min.

All PCRs were run on an Eppendorf Mastercycler (Eppendorf AG, Hamburg, Germany) with an initial hot start at 94 °C for 10 min and a final extension at 72 °C for 7 min. Amplicons were visualized by 2% agarose gel electrophoresis stained with GelRed™ stain (BioTrend, Cologne, Germany), cut from the gel with a sterile scalpel and purified using the PCR and Gel Band Purification kit (illustra GFX, GE Healthcare, Austria). Sanger sequencing was performed directly from the PCR products with a Thermo Fisher Scientific SeqStudio (Thermo Fisher Scientific, MA, USA). Sequences were obtained from both strands in two independent setups, aligned to obtain a consensus sequence using ClustalW in BioEdit Sequence Alignment Editor [32] and compared using NCBI BLAST to reference sequences of *Cryptosporidium*, *Giardia* and *Eimeria* species and genotypes from GenBank [33].

Representative sequences for each parasite species, genotype, and assemblage generated from this study have been deposited in GenBank under the following accession numbers: *Cryptosporidium* (MZ314966–75), *Eimeria* (MZ314986–92) and *Giardia* (MZ322740–51 and MZ393409).

### 2.5. Statistical Analysis and Mapping

Maps of the capture sites were built using QGIS 3.4.15 (QGIS Development Team, 2018); the raster dataset “Orthofoto 2016 Wien” (Open Data Österreich, https://www.data.gv.at/, 31 March 2019) was used as base map. Statistical analysis was performed as described in [27]. Briefly, the spatial autocorrelation between parasite positive rats was assessed using a non-parametric spatial covariance function. We investigated the impact of place of capture, body mass, sex, sexual maturity and land-use variables (within a 200 m radius buffer zone around place of capture, considered as a proxy for home range) on the individual infection status of rats for the three parasites.

We computed a logistic regression model (generalized linear mixed-effect model under binomial distribution) using the *glmer* function in the *lme4* library while controlling for clustering by site of capture (random effect). The conditional model average estimates were calculated and weighted according to the Akaike Information Criterion corrected for small sample size (AICc). For each covariate, the relative variable importance (RVI) was computed from model averaged parameter estimate weights to determine the probability that each variable might contribute to the model for these data.

### 2.6. Ethical Statement

This study followed institutional and national standards for the care and use of animals in research. It was approved by the institutional ethics and animal welfare committee and the national authority (GZ 68.205/0196-WF/V/3b/2016).

## 3. Results

### 3.1. Trapping

**Figure 1:**
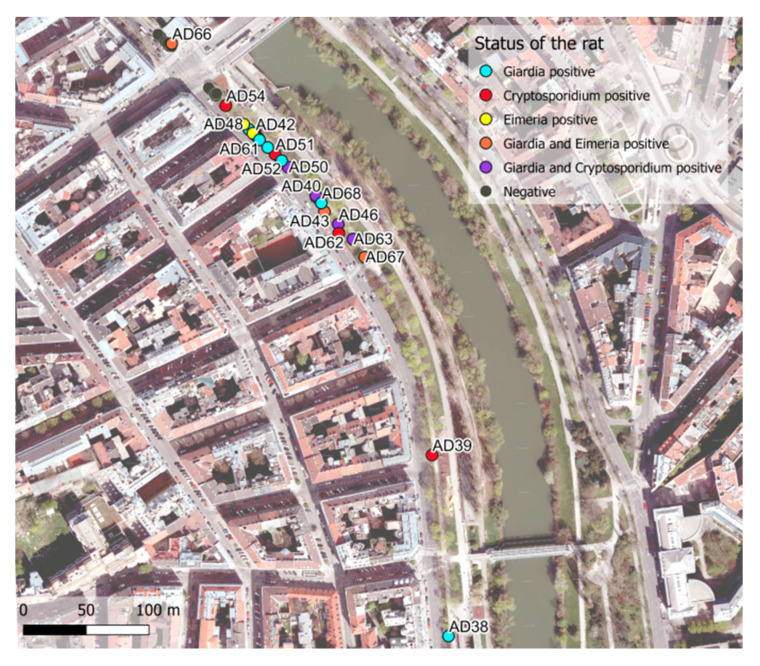
Spatial distribution of the trapped urban brown rats (*R. norvegicus*) at the Danube Canal, March-June 2017, Vienna, Austria. Parasite-positive rats are labeled. Rats trapped were sequentially named AD31–84.

**Figure 2:**
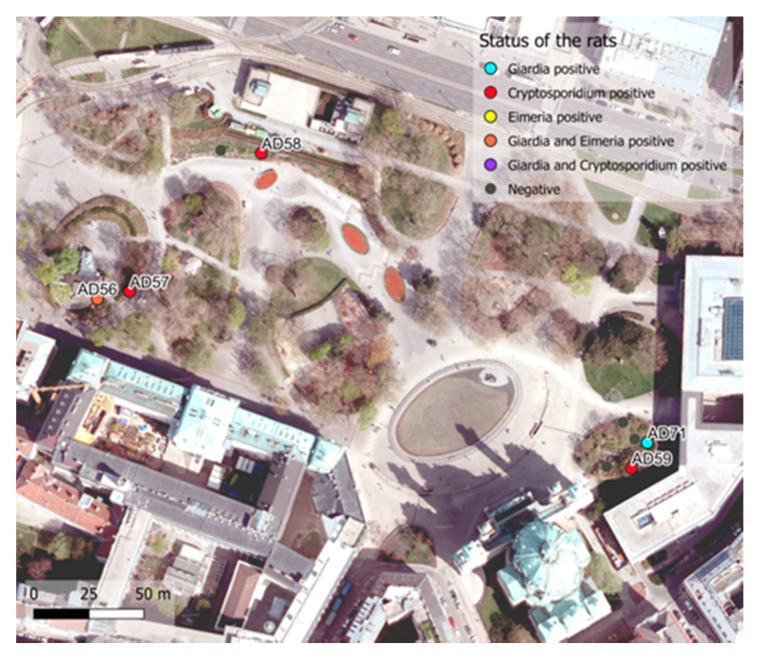
Spatial distribution of the trapped urban brown rats (*R. norvegicus*) at Karlsplatz, April-June 2017, Vienna, Austria. Parasite-positive rats are labeled. Rats trapped were sequentially named AD31–84.

Traps were set on 15 nights in two locations in Vienna (10 at Danube Canal, five at Karlsplatz) and 50 brown rats (*R. norvegicus*) were captured (Danube Canal 39; Karlsplatz 11) (Figure 1 and Figure 2). Twenty-eight (56%) of the captured rats were male (Danube Canal 23; Karlsplatz 5) and 22 (44%) were female (Danube Canal 16; Karlsplatz 6). Among them, 24 (48%) were sexually mature (15 male and 9 female). The median body mass and length (nose tip to anus) were 129.5 g and 167.5 mm for rats caught at Danube Canal, and 91.4 g and 153 mm at Karlsplatz. The median body mass of sexually mature rats was 176.8 g versus 121.1 g for immature rats (Table 1, Table S1).

### 3.2. Prevalence and Identification of Protozoa in Rat Faeces

Overall, 74% (*n* = 34) of the stool samples from the 50 urban brown rats analyzed by independent nested PCR provided *Giardia* and/or *Cryptosporidium* sequences. Interestingly, *Eimeria* was also detected by the *Cryptosporidium* nested PCR. Co-occurrence of *Cryptosporidium* and *Giardia* was observed in six rats and co-occurrence of *Eimeria* and *Giardia* was observed in four rats (Figure 1, Figure 2, Table 1, Table S1).

We obtained a limited amount of DNA from the rat feces; thus, we aimed to sequence every sample at least twice in independent setups to obtain reliable consensus sequences, and we concentrated our efforts on identifying to the species/assemblage level the three protozoa shed by rats in the present study rather than exploring in depth the phylogeny of only one of them. Thus, no further testing regarding *Cryptosporidium* and *Eimeria* species as well as *Giardia* sub-assemblages was possible.

#### 3.2.1. Cryptosporidium

Of the 50 rat fecal samples analyzed, 20 (40%) revealed an amplicon in the 18S rDNA nested PCR for *Cryptosporidium*. Seventeen of these 20 samples were successfully sequenced. The sequence analysis identified 10 of them belonging to the genus *Cryptosporidium* and seven belonging to the genus *Eimeria*.

Among the *Cryptosporidium* sequences, we identified five sequences (MZ314966–70) with 100% similarity to *Cryptosporidium* rat genotypes I and IV available in GenBank and previously isolated from rodents, specifically rats. The fragment of the 18S rDNA analysed here shows no nucleotide differences between the two genotypes to further distinguish between them. We also identified four sequences (MZ314971–74) with 99.81–100% similarity to others found in GenBank identified as *Cryptosporidium* environmental spp. and isolated from wastewater (KY483983), storm water (AY737582, AY7375824 and AY7375825) and brown rats (MT56130, MG917671, MT504540) from all over the world. Moreover, we identified one sequence (MZ314975) with 100% sequence identity to those reported in [34] and in recently named *C. occultus* n. sp.

#### 3.2.2. Eimeria

The analysis of the 18S rDNA amplicons revealed that seven sequences belonged to the genus *Eimeria*. Among the seven sequences, five of them (MZ314986–90) are 99.82–100% identical to the sequences of *E. alorani* (MK625209, KU192965) and *E. caviae* (JQ993649) available in GenBank and isolated from other rodents. No further species distinction was possible due to the low sequence diversity of these two species in the amplified region (1 bp) and there being only one 18S rDNA *E. caviae* sequence available in GenBank. The other two sequences obtained (MZ314991–92) show 100% identity to *E. ferrissi* sequences available in GenBank and isolated from mice (MH752036, MH751925) and squirrels (KT360995).

#### 3.2.3. Giardia

Of the 50 rat fecal samples analyzed by nested PCR targeting the *tpi* gene locus of *Giardia*, 17 (34%) gave amplicons of the correct length. Of these 17 amplicons, 12 were successfully sequenced, revealing two *Giardia* assemblages, namely assemblage G (*n* = 3) and assemblage A (*n* = 9). Two of the *Giardia* assemblage G sequences obtained (MZ322740–41) were 100% identical to others available in GenBank isolated from rats (MT114179, EU781013), whereas sequence MZ322742 showed one nucleotide difference. Among the nine sequences belonging to assemblage A, eight sequences MZ322743–50 showed 100% identity to sequences from GenBank classified as assemblage AI and isolated from cattle (EF654693) and sheep (MK639171). Moreover, sequence MZ322751 showed a 99.8% similarity with other sequences available in GenBank classified as assemblage AII and isolated from donkeys (MN704937), dogs (KY608997) and cats (LC341572) from all over the world.

### 3.3. Land-Use Data

For each investigated site, the surface occupied by each land-use category in a 200-m radius buffer area (proxy for home range) of the captured rats is summarized in Table 2.

### 3.4. Predictors of Cryptosporidium, Eimeria and Giardia Shedding in Urban Rats

The non-parametric spatial correlation did not reveal a spatial correlation between rats trapped and the shedding of the three investigated protozoan parasites (Table S2, Supplementary Material). Statistical analyses showed that the investigated variables (place of capture, body mass, sex, sexual maturity and land-use) were low to moderate predictors of *Cryptosporidium*, *Eimeria* or *Giardia* shedding. The variable that contributed the most to predicting the shedding of *Cryptosporidium* by brown rats was the presence of green infrastructure in the rats’ home range (RVI 0.60); the most important predictor of *Eimeria* shedding was sexual maturity, although the RVI was low (0.31), and the presence of transport infrastructure in the rat home range was found to be the most important predictor of *Giardia* shedding (RVI 0.83) (Supplementary Material, Table S3).

### 3.5. Prevalence and Identification of Cryptosporidium and Giardia in Surface Water Samples

*Cryptosporidium* was detected in nine of the 15 water samples analysed between May 2019 and September 2020, in concentrations ranging from 0.3–4 oocysts/L. *Giardia* was detected in 11 samples, in concentrations ranging from 0.6–96 cysts/L (Table 3). *Cryptosporidium* and *Giardia*’s highest concentrations from November 2019 occurred during a rainy day, however we have no explanation for the unusual high values shown in May 2020.

Volumes of surface water filtered were determined by the turbidity of the water samples. Samples with high turbidity limited the filtration to 5–7 L in some cases. Due to the variability of volumes filtered, sample-specific limits of detection were calculated (Table 3).

Out of 15 samples, 12 revealed an amplicon in the 18S rDNA nested PCR for *Crypto-sporidium.* Of these 12 samples, only six generated a good consensus sequence; however, none showed >97% sequence similarity with any *Cryptosporidium* species available in GenBank. Four of them were identified as a *Perkinsea* species known to be a frog parasite, and one as *Peridinopsis penardii*, a dinoflagellate. Both of these genera also belong to the alveolates, as *Cryptosporidium* does, and both are known to be abundant in fresh water samples. One other sample showed the highest sequence identity to an unidentified stramenopile, the stramenopiles being the sister taxon of the alveolates.

Regarding *Giardia*, out of 15 samples we successfully sequenced one amplicon of the *tpi* gene locus of *Giardia* spp. The 295 bp long sequence (MZ393409) had a 100% similarity with various *Giardia* assemblage C sequences found in GenBank.

## 4. Discussion

The present study reports for the first time the prevalence of *Cryptosporidium*, *Eimeria,* and *Giardia* at the wildlife–water interface ecosystem in two densely populated sites located in the city center of Vienna, the capital of Austria.

*Cryptosporidium* rat genotypes I/IV and *Cryptosporidium* environmental species identified in our study have been previously isolated from brown rat feces worldwide [35] showing a good capacity of these *Cryptosporidium* genotypes for infecting rats [35]. *C. occultus* has also been previously isolated in domesticated animals, brown rats, and once in humans; however, it failed to infect calves under controlled experimental conditions [34]. Our results showed that at the time they were trapped, urban brown rats did not shed zoonotic *Cryptosporidium* species. Although the prevalence of *Cryptosporidium* was very similar in rats trapped at the Danube Canal and Karlsplatz, at the latter only *Cryptosporidium* rat genotype I/IV sequences were identified. Differential pathogen prevalence and the existence of isolated patches among rats within the same city has also been reported in other studies [36,37,38]. Such differences have mostly been attributed to the fragmentation of the urban environment.

The nested PCR used to sequence *Cryptosporidium* also detected *Eimeria* sequences. That fact can be explained by the high similarity of the two genomes, classified within the same taxonomical Order *Eucoccidioida*. Coccidians of the genus *Eimeria* have been described as host-specific intracellular parasites [39]. Several members of the genus cause considerable morbidity and mortality in livestock and wildlife and are thus of veterinary importance [40]. *Eimeria* species associated with rodents show a degree of host specificity, but individual isolates can experimentally infect different species and even genera of rodents [39]. Recent studies on rural rodents in Central Europe reported an *Eimeria* prevalence of 32.7% combining coprological investigations with molecular methods [41]. In our study, without using specific primers for *Eimeria* spp., we detected the parasite in 14% of rats trapped. Out of the seven amplicons obtained, five showed a 99–100% identity with the 18S rDNA sequences of *E. alorani* and *E. caviae* isolated from other rodents and the other two amplicons obtained were 100% identical with sequences of *E. ferrissi* isolated from mice [41]. In fact, all molecular studies on the prevalence of *Eimeria* in rodents from urban or rural areas performed in the past 20–25 years have focused on mice. The diversity of *Eimeria*, as observed for *Cryptosporidium,* differed according to the site, being lower at the Karlsplatz than the Danube Canal, supporting the hypothesis of the existence of isolated patches due to the fragmentation of the urban environment [36,37]. Regardless of that, our results suggest that Viennese brown rats are infected by rodent-specific *Eimeria* species, thus spillover to other domestic animals such as dogs and cats seems unlikely.

Among the 50 rat feces samples analysed for *Giardia* in our study, the *tpi* locus was successfully sequenced for 12 samples revealing two *Giardia* assemblages, namely the typical rodent assemblage G (*n* = 3) and the typically human but zoonotic assemblage A (*n* = 9).

*Giardia* assemblage G sequences obtained in our study had 99.8–100% similarity to other sequences isolated from rats [42,43], which is not surprising since rats are considered the major hosts of that assemblage [44]. Among the nine sequences belonging to assemblage A, eight showed 100% identity to sequences classified as assemblage AI and isolated from sheep [45], cattle [46] and humans [47]. The other isolate belonging to assemblage A showed a 99.8% similarity with sequences classified as assemblage AII, previously isolated from other mammals [48] and, as observed with assemblage AI sequences, humans [49]. Among the sequences available in GenBank that had a 100% similarity with the latter assemblage A sequence obtained in our study, only one had been previously isolated from rodents, specifically from prairie dogs [50]. Thus, our study is the first one reporting identical *Giardia* assemblage A isolate in rats that has elsewhere caused symptomatic infections in humans, suggesting that *Giardia* shed by rats in Vienna may pose a risk for public health.

Co-occurrence of two protozoa parasites was observed in ten rats, with six of them shedding *Cryptosporidium* and *Giardia* and four of them shedding *Eimeria* and *Giardia*. Both protozoa were successfully sequenced in five rats. Most of the *Cryptosporidium* and *Eimeria* species as well as *Giardia* assemblage G isolated from urban brown rats’ feces in the city center of Vienna are known to infect rats or other rodents. However, *Giardia* assemblage A, shed by the majority of the rats in our study, has the broadest host-range, infecting all kinds of mammals including livestock, cats, dogs, rodents, marsupials, non-human primates and humans [8]. Thus, although human-to-human infections are common, assemblage A-related infections can have a zoonotic origin. In our study, the prevalence of rats shedding *Giardia* was higher at the Danube Canal (38% vs. 18%). As rats were trapped within a <200 m radius of the Danube Canal, we hypothesized that *Giardia* assemblage A sequences may have been transmitted via water.

*Cryptosporidium* and *Giardia* (oo)cysts were detected in 60% and 73% of water samples taken <1 km downstream from where the rats were trapped at the Danube Canal with concentrations ranging between 0.3–4 oocysts/L and 0.6–96 cysts/L (median 1 (oo)cyst/L), respectively.

The occurrence of *Cryptosporidium* and *Giardia* in surface waters has been traditionally linked to seasonality and more specifically to rainfall events worldwide [51]. This information needs to be taken into account when comparing studies conducted during different seasons and years. From the most upstream point of the Danube basin to its mouth, *Cryptosporidium* oocysts have been detected in 40% of the samples taken monthly over 2004–2005 downstream from Budapest (Hungary) before the implementation of a wastewater treatment plant, in concentrations ranging from 0–0.5 oocysts/L [52], detected but not quantified in Serbia [53], and detected from June to September at the river mouth in Romania in 2010 with concentrations ranging from 10–65 oocysts/L [54]. *Giardia* cysts were frequently detected with concentrations ranging from 1.35–3 cysts/L at the same spot in Hungary [52] over 2004–2005, detected but not quantified in two different spots within Serbia without co-occurrence with *Cryptosporidium* [53], and also detected from June to September at the river mouth in Romania in 2010 with concentrations ranging from 4–45 cysts/L [54]. As in our results, *Giardia* concentrations reported by the aforementioned studies were usually higher than *Cryptosporidium*, except at the river mouth. Although the concentration of both parasites may seem low in comparison to the traditional standard fecal indicator bacteria used to assess the quality of recreational waters, note that the infective stages of both parasites’ cysts and oocysts are robust and able to survive longer than bacteria under harsh environmental conditions and disinfectants commonly used for the production of drinking water, such as chlorine, as well as wastewater treatments [55]. Moreover, they have a very low infectious dose—as few as ten *Giardia* cysts or one *Cryptosporidium* oocyst may be enough to cause infection [3,55]. Despite the fact that in none of the studies aforementioned, including the present one, was the infectivity of the parasites tested, our results highlight the need for more environmental studies for a correct assessment of the risk of protozoan infections while using the River Danube for recreational purposes or for drinking water production.

The low concentration of (oo)cysts detected in most of the water samples, the presence of numerous species/genotypes co-occurring in the same sample thus making identification by direct sequencing without cloning impossible, and the rich diversity and partly high abundance of other, related protists in freshwater (e.g., other alveolates or excavates), hindered the identification below the genus level of many samples. Moreover, *Cryptosporidium* oocysts may remain in the environment as empty shells, so-called “ghosts”, after losing their nuclei and with them their genetic information. Nonetheless, they can still be detected and quantified by the flat membrane method used in this study. Regarding *Giardia* taxonomy, there are also several hurdles one has to overcome when identifying isolates, among the most important ones being co-occurrence of more than one genotype, potential recombination within a population, allelic sequence heterozygosity (ASH), specificity of the primers used which bind preferentially to certain genotypes, and non-concordance between loci [5,15]. Thus, the identification of *Cryptosporidium* and *Giardia* species and assemblages from (oo)cysts found in environmental sources pose a real challenge.

During the Joint Danube Survey of 2013, a six week monitoring campaign on Danube water quality, Kirschner et al. [1] demonstrated that the major contributors to the microbial fecal pollution of the Danube were humans. Other contributors such as ruminants and pigs were detected in <10% of the samples with low concentrations, despite animal farming and pastureland along the river [1]. According to these findings, a majority of *Cryptosporidium* and *Giardia* species and assemblages typically infecting humans such as *C. hominis* and *C. parvum* from both human and zoonotic origin, as well as *Giardia* assemblages A and B, would be expected. In our study we tried to identify species, genotypes and assemblages of the two parasites; however, all sequences obtained with the primers specific for *Cryptosporidium* were in fact from other alveolates, indicating less than 100% specificity for these primers and a higher density of these other alveolates in the water samples investigated. Similarly, for *Giardia*, only one isolate was sequenced successfully, revealing *Giardia* assemblage C, the “dog” genotype, the source of which could be close as a result of urban run-off or much further upstream.

In Austria, there is no information on the number of infections caused by *Cryptosporidium* and *Giardia*, since they are both not reportable diseases. Germany, with a population 10 times higher, reported 1974 *Cryptosporidium* and 3296 *Giardia* infections in 2019 [56]. Lee et al. [57] determined the assemblages of *Giardia* causing diarrhea in Austrian patients in 2015. Interestingly, 65.4% of the infections were caused by assemblage B, whereas 34.6% of them by assemblage A, among which 25% were classified as sub-assemblage AII and 9.6% of them as sub-assemblage AI. Authors suggested that the high diversity found among *Giardia* isolates could be explained by travelers returning from various endemic areas worldwide [57].

The urban brown rats trapped in Vienna in 2017 and part of the Austrian patients with diarrhea in 2015 shed the same zoonotic *Giardia* genotypes. Our study did not reveal proximity to water as a predictor for rats shedding *Giardia*. Moreover, the *Giardia* assemblage identified at the Danube Canal was assemblage C. The number of rats trapped in the present study does not provide strong statistical power to compute a robust model, and rats were trapped in 2017 whereas water samples were taken over 2019–2020. Moreover, *Cryptosporidium* and *Giardia* are known to seasonally infect humans and thus, their presence in wastewater and surface water also varies over the year [51].

Therefore, given the presence of *Giardia* assemblages infecting urban brown rats and humans within the same urban environment, it is crucial to monitor the prevalence and species diversity of protozoan parasites in humans and their reservoirs. Moreover, elucidating potential transmission pathways such as contact with wastewater or fecally polluted surface waters will help in detecting potential emerging threats for public health and designing effective preventive strategies within a One Health approach. Such an approach may include an integrated pest management program taking into account the ecology of urban rats [17,27].

## Figures and Tables

**Table 1:** Prevalence of *Cryptosporidium*, *Eimeria,* and *Giardia* in the faeces of urban brown rats (*R. norvegicus*) calculated according to rat and trapping characteristics.

		Prevalence (%)		
Variable	*n*° Rats	*Cryptosporidium*	*Eimeria*	*Giardia*
Sex				
Male	28/50 (56%)	6/28 (21.4%)	4/28 (14.3%)	10/28 (35.7%)
Female	22/50 (44%)	7/22 (31.8%)	3/22 (13.6%)	7/22 (31.8%)
Age				
Sexually immature	26/50 (52%)	5/26 (19.2%)	5/26 (19.2%)	8/26 (30.8%)
Sexually mature	24/50 (48%)	8/24 (33.3%)	2/24 (8.3%)	9/24 (37.5%)
Body mass				
0–200 g	29/50 (58%)	5/29 (17.2%)	6/29 (20.7%)	8/29 (27.6%)
>200 g	21/50 (42%)	8/21 (38.1%)	1/21 (4.8%)	9/21 (42.8%)
Body length (nose to anus)				
100–200 mm	32/50 (64%)	7/32 (21.9%)	6/32 (18.7%)	9/32 (21.9%)
>200 mm	18/50 (36%)	6/18 (33.3%)	1/18 (5.5%)	8/18 (44.4%)
Trapping month				
March	23/50 (46%)	8/23 (34.8%)	4/23 (17.4%)	9/23 (39.1%)
April	9/50 (18%)	5/9 (55.5%)	1/9 (11.1%)	3/9 (33.3%)
May	8/50 (16%)	0/8 (0%)	2/8 (25%)	5/8 (62.5%)
June	10/50 (20%)	0/10 (0%)	0/10 (0%)	0/10 (0%)
Trapping site				
Danube Canal	39/50 (78%)	10/39 (25.6%)	6/39 (15.4%)	15/39 (38.5%)
Karlsplatz	11/50 (22%)	3/11 (27.3%)	1/11 (9.1%)	2/11 (18.2%)

**Table 2:** Land-use categories within the rat home range (based on a 200 m-radius buffer surface around each capture point) shown in average percentage for the two trapping sites in Vienna, Austria.

Trapping Site	Transport	Building	Green Infrastructure	Blue Infrastructure
Danube Canal	29.3%	30.6%	25.7%	14.4%
Karlsplatz	32.9%	41.4%	25.7%	0%

**Table 3:** Sampling date, volume filtered, turbidity and *Cryptosporidium* and *Giardia* (oo)cyst concentration/L at the Danube Canal from May 2019 until September 2020. The sample limit of detection (<) is given when no *Cryptosporidium* and *Giardia* (oo)cyst were observed.

Sampling Date	Volume Filtered (L)	*Turbidity (NTU)*	*Cryptosporidium* Oocysts/L	*Giardia* Cysts/L
7 May 2019	10	23	3	1
4 June 2019	5	67	4	0.8
9 July 2019	7	70	<0.6	<0.6
6 August 2019	7	29	<0.6	0.6
3 September 2019	7	24	<0.6	2.9
8 October 2019	10	16	1	1
5 November 2019	10	11	2.8	57.6
10 December 2019	10	14	1.6	1.2
14 January 2020	10	5.8	1.2	3.2
24 February 2020	10	14	<2	<2
5 May 2020	10	10.5	4	96
9 June 2020	10	10	<0.4	1.2
7 July 2020	5	80	<0.8	0.8
11 August 2020	7	35	2.9	<0.6
22 September 2020	15	13	0.3	<0.3

## Data Availability

The data presented in this study are available in the Supplementary Material files (Tables S1–S3). Sequences for each parasite species, genotype, and assemblage generated from this study have been deposited in GenBank under the following accession numbers: *Cryptosporidium* (MZ314966-75), *Eimeria* (MZ314986-92) and *Giardia* (MZ322740-51 and MZ393409).

## Supplementary Materials

The following are available online at https://www.mdpi.com/article/10.3390/microorganisms9081596/s1, Table S1: Dataset: characteristics of the rats (*Rattus norvegicus*) captured for this study, Vienna, Austria, 6 March–20 June 2017; Table S2: Recovery efficiencies of the flat membrane method used for the enumeration of *Cryptosporidium* and *Giardia* from surface water samples; Table S3: Model averaged variable estimates (conditional average), adjusted standard errors (SE) and *p values* of the generalized mixed linear model of *Cryptosporidium*, *Eimeria* and *Giardia* shedding in rat feces with binomial distribution and logit link function. The contribution of each variable to the model is shown by the relative variable importance (RVI) values.
