# Supplementary material for: Surface Waters and Urban Brown Rats as Potential Sources of Human-Infective Cryptosporidium and Giardia in Vienna, Austria
Source: Microorganisms. 2021 Jul 27;9(8):1596. doi: 10.3390/microorganisms9081596 (PMC8400309; doi:10.3390/microorganisms9081596)
Supplement: Supplementary file 1 [file microorganisms-09-01596-s001.zip › Supplement_mat_TableS2_S3.pdf]

Surface waters and urban brown rats as potential sources of human-infective *Cryptosporidium* and *Giardia* in Vienna, Austria

Silvia Cervero-Aragó*[1,2], Amélie Desvars-Larrive[3,4,5], Gerhard Lindner[1,2], Regina Sommer[1,2], Iveta Häfeli[6], Julia Walochnik[6]

[1] Institute for Hygiene and Applied Immunology, Medical University of Vienna, 1090 Vienna, Austria; silvia.cerveroarago@meduniwien.ac.at
[2] Interuniversity Cooperation Centre Water & Health (www.waterandhealth.at)
[3] Unit Veterinary Public Health and Epidemiology, University of Veterinary Medicine, 1210 Vienna, Austria; Amelie.Desvars@vetmeduni.ac.at
[4] VetFarm, University of Veterinary Medicine, 1210 Vienna, Austria
[5] Complexity Science Hub Vienna, Austria
[6] Institute of Specific Prophylaxis and Tropical Medicine, Medical University of Vienna, 1090 Vienna, Austria; julia.walochnik@meduniwien.ac.at
* Correspondence: silvia.cerveroarago@meduniwien.ac.at; Tel.: +43-1-40160-33059

Table S2. Recovery efficiencies of the flat membrane method used for the enumeration of *Cryptosporidium* and *Giardia* from surface water samples. For each test the date, type of water matrix, volume filtered, turbidity and the percentage of the recovery efficiencies of both protozoa are given. NA (not analyzed).

Spiking date	Water matrix	Volume filtered (L)	Turbidity (NTU)	Recovery efficiency (%)	
				Cryptosporidium oocysts	*Giardia* cysts
18/04/2018	Danube	1 L	NA	NA	96
				NA	67
				NA	97
02/05/2018	New Danube	10 L	1.7	NA	100
				NA	67.6
				NA	73
09/05/2018	New Danube	10 L	2.3	NA	29.6
				NA	88.89
				NA	59.26
12/12/2018	Danube	5 L	32	74.34	97.1
				60.73	100
11/06/2019	Danube Canal	5 L	73	31	33.48
				30.22	33.48
				28.4	48.37
24/08/2020	Danube	5 L	70	69.7	55.5
				32.17	15.38
				100	47
**Geom. Mean ± SD**				**48 ± 27**	**58 ± 28**

Table S3. Model averaged variable estimates (conditional average), adjusted standard errors (SE) and *p values* of the generalized mixed linear model of *Cryptosporidium*, *Eimeria* and *Giardia* shedding in rat faeces with binomial distribution and logit link function. The contribution of each variable to the model is shown by the relative variable importance (RVI) values.

		Model average estimates (adjusted SE)	p value	RVI
Cryptosporidium	Intercept	-2.93 (5.77)	0.611	
	Body mass	0.001 (0.002)	0.650	0.35
	Sex (Male)	-0.13 (0.40)	0.739	0.28
	Sexual maturity (mature)	0.11 (0.48)	0.819	0.28
	Blue infrastructure	-0.008 (0.03)	0.812	0.26
	Green infrastructure	0.13 (0.14)	0.365	0.60
	Transport infrastructure	-0.04 (0.09)	0.666	0.37
Eimeria	Intercept	-0.72 (3.70)	0.846	
	Body mass	-0.0003 (0.002)	0.887	0.26
	Sex (Male)	-0.02 (0.34)	0.962	0.23
	Sexual maturity (mature)	-0.21 (0.60)	0.719	0.31
	Blue infrastructure	0.004 (0.03)	0.891	0.23
	Green infrastructure	-0.01 (0.08)	0.892	0.24
	Transport infrastructure	-0.009 (0.06)	0.890	0.24
Giardia	Intercept	5.74 (7.18)	0.424	
	Body mass	0.000006 (0.002)	0.997	0.23
	Sex (Male)	0.03 (0.30)	0.923	0.23
	Sexual maturity (mature)	-0.03 (0.36)	0.926	0.23
	Blue infrastructure	0.003 (0.03)	0.933	0.26
	Green infrastructure	-0.02 (0.13)	0.898	0.31
	Transport infrastructure	-0.19 (0.14)	0.175	0.83
